# ACT001 improves OVX-induced osteoporosis by suppressing the NF-κB/NLRP3 signaling pathway

**DOI:** 10.1186/s10020-025-01189-3

**Published:** 2025-04-07

**Authors:** Yuan Li, Jin-Yu Yang, Ma-Li Lin, Tian-Zhu Liu, Wen-Na Lu, Ying Yang, Zhong-Cheng Liu, Jian-Heng Li, Guo-Qiang Zhang, Jian-Shuang Guo

**Affiliations:** State Key Laboratory of New Pharmaceutical Preparations and Excipients, Key Laboratory of Medicinal Chemistry and Molecular Diagnosis of Ministry of Education, College of Pharmaceutical Sciences, Hebei University, Baoding, 071002 China

**Keywords:** ACT001, Osteoporosis, Osteoclastogenesis, NF-κB pathway, NLRP3 inflammasome

## Abstract

Osteoporosis (OP) is a common systemic metabolic bone disease characterized by the decrease in bone mass and hyperactivity of osteoclasts. ACT001 is approved as an orphan drug by FDA and has shown multiple protective effects against tissue injury. However, its role in prevention of osteoclast differentiation and the underlying mechanisms have not been elucidated. Herein, we show that ACT001 inhibited RANKL-induced osteoclast differentiation and F-actin ring formation through suppressing the expression of Nfatc1, TRAP, Ctsk, Dc-stamp without obvious cytotoxicity in vitro. ACT001 restrained the phosphorylation of NF-κB and the activation of NLRP3 inflammasome, thereby decreased the expression of pyroptosis-related protein. (GSDMD, caspase-1, IL-1β, IL-18). Consistent with ACT001, the NLRP3 inflammasome inhibitor MCC950 treatment also suppressed the osteoclastogenesis through inhibiting the transcriptional activation of Nfatc1. Furthermore, ACT001 protected ovariectomy-induced bone loss in mice, reduced the number of osteoclasts, downregulated the expression of NLRP3 and IL-1β. These data indicate that ACT001 can reduce RANKL-induced osteoclast differentiation through suppressing the NF-κB/NLRP3 pathway, and attenuate the bone loss induced by estrogen-deficiency, suggesting its therapeutic potential for bone homeostasis maintenance and osteoporosis treatment.

**Supplementary Information:**

The online version contains supplementary material available at 10.1186/s10020-025-01189-3.

## Introduction

As the increasing aging populations, osteoporosis is becoming one of the most common metabolic bone diseases that is characterized by changes in bone homeostasis, which lead to reduced bone mass and increased bone fragility fractures (Li et al. 2021). There are over 200 million people suffering from osteoporosis and over 50% of postmenopausal women were subjected to osteoporotic-associated fracture, which severely compromise physical functioning and quality of life ((2016) 2016). Currently, several pharmacological agents are available for the treatment of osteoporosis, but there are still some own limitations and side effects in long-term treatment, such as hypercalcemia, mandibular necrosis, atypical femoral fracture, and so on (Reid and Billington 2022; Liang et al. 2022). Therefore, more effective anti-osteoporosis treatment strategies will to be developed continuously.

Estrogen deficiency is the main pathogenetic factors of osteoporosis, which increases the activation of nuclear factor kappa-Β ligand (RANKL) and decrease the production of OPG by osteoblasts and lymphocytes, thus upregulated the RANKL/OPG ratio and imbalanced the bone remodeling, mainly manifested as the imbalance of the activity of osteoblasts and osteoclasts (Gao et al. 2021; Zhivodernikov et al. 2023). Osteoclasts are mainly involved in bone resorption, which is influenced by hormones, cytokines, and noncoding RNAs, and can lead to osteoporosis by activating signaling pathways related to osteoclast differentiation (Adejuyigbe et al. 2023; Kenkre and Bassett 2018). Of which, receptor activator of RANKL secreted by osteocytes can bind the RANKL-specific receptor (RANK) on osteoclasts, which then recruit tumor necrosis factor receptor-related factor-6 (TRAF6) to trigger nuclear factor kappa-Β (NF-κB), phosphatidylinositol 3-kinase (PI3K), mitogen-activated protein kinase (MAPK) signaling pathway (Liang et al. 2022). This results in the activation of nuclear factor of activated T cells-1 (NFATc1) and so on, and regulates osteoclast activation, maturation, or differentiation (Takegahara et al. 2022). Thereby inhibiting RANKL/RANK and its downstream signaling pathways will be the most promising options against osteoclastogenesis (Liu et al. 2020; Udagawa et al. 2021).

Recent findings demonstrate that chronic inflammatory conditions induced by estrogen deficiency can activate NOD-like receptor family pyrin domain-containing 3 (NLRP3) inflammasome, which impairs bone formation and promotes bone resorption, and inhibiting the activation of NLRP3 inflammasome may prevent or control the progression of osteoporosis (Liang et al. 2020; Tao et al. 2021). ACT001 has been certified as an orphan drug by the U.S. Food and Drug Administration and in the European Union and has multiple biological activities, including anti-tumor (Tong et al. 2020), anti-inflammatory (Guo et al. 2022a, b), neuroprotective (Liu et al. 2020) and more (Luo et al. 2023). Currently, several clinical trials of ACT001 for treatment of cancer are ongoing in China and Australia. ACT001 is the fumarate salt form of dimethylamino micheliolide (DMAMCL), which is developed from micheliolide (MCL), a guaianolide sesquiterpene lactone (Guo et al. 2022a, b). Previously studies have reported that ACT001 can attenuate neuroinflammation after traumatic brain injury via inhibiting AKT/NF-κB/NLRP3 pathway (Cai et al. 2022), and also inhibit glioblastoma cell growth by targeting IKKβ and inhibiting NF-κB activation (Li et al. 2020). Moreover, ACT001 alleviates inflammation and ionizing radiation-induced lung injury through the NF-κB and NLRP3 signaling pathway (Luo et al. 2023). ACT001 can also attenuates neuropathic pain by restraining the activation of TLR4 signaling axes of NF-κB and MAPKs through targeting co-receptor MD2 (Zhang et al. 2022). However, the preventive effects of ACT001 on osteoporosis are still unclear. In this study, we investigated the inhibiting effects and mechanisms of ACT001 on osteoclast differentiation in vitro. And the protective effect of ACT001 for postmenopausal osteoporosis was further verified by establishing ovariectomy (OVX)-induced osteoporosis mice model in vivo.

## Materials and methods

### Chemicals and reagents

ACT001 was provided by Accendatech Co.,Ltd (Tianjin, China). MCC950 and MD2-IN-1 were purchased from MedChemExpress (Shanghai, China). Recombinant RANKL was purchased from novoprotein (Suzhou, China). CCK-8 kit was purchased from Abbkine (Wuhan, China). Tartrate resistant acid phosphatase (TRAP) staining kit, Alexa Fluor 488-phalloidin, toluidine blue, Alizarin red S (ARS) Solution (1%, pH 4.2) were purchased from Servicebio (Wuhan, China). β-actin monoclonal antibody (66009-1-Ig) and Nfatc1 monoclonal antibody (66963-1-Ig) were purchased from Proteintech (Wuhan, China). Ctsk polyclonal antibody (A1782), pJNK monoclonal antibody (AP0631), JNK monoclonal antibody (A4867), p38 polyclonal antibody (A14401), p-p38 polyclonal antibody (AP0526) and NLRP3 polyclonal antibody (A5652), Caspase-1 polyclonal antibody (A0964), IL-18 polyclonal antibody (A1115) and GSDMD polyclonal antibody (A18281) were purchased from Abclonal (Wuhan, China). ERK monoclonal antibody (3192), p-ERK monoclonal antibody (3179), p65 monoclonal antibody (8242) and p-p65 monoclonal antibody (3033) were purchased from Cell Signaling Technology (Danvers, MA, USA). IL-1β monoclonal antibody (sc-12742) was purchased from Santa Cruz Biotechnology (Dallas, TX, USA). Alexa Fluor 488-labeled Goat Anti-Mouse IgG (H + L) and BCIP/NBT Alkaline Phosphatase (ALP) Color Development Kit were purchased from Beyotime (Shanghai, China). PINP, BALP, CTX-1, TRAP ELISA kit (2808, 2776, 2814, 2775) was purchased from Shfksc (Shanghai, China).

### Cell culture

RAW264.7 cell and MC3T3-E1 cell were obtained from the Cell Bank of the Chinese Academy of Sciences (Shanghai, China). Cells were cultured in α-MEM (Corning, Manassas, VA, USA) supplemented with 10% FBS (Sigma-Aldrich, Missouri, USA) and 1% (v/v) penicillin/streptomycin (Gibco Life Technologies, Grand Island, NY, USA) at 37 ^o^C, 95% humidity and 5% CO_2_.

### Isolation of bone marrow-derived macrophages (BMMs)

Using 4-6-week-old C57BL/6J mice, the femurs and tibias were excised and muscle tissues were removed. The epiphyses were severed, and bone marrow cavities were flushed with α-MEM medium. The cell suspension was centrifuged (1200 rpm, 3 min) and resuspended in complete α-MEM medium (containing 10% FBS, 1% PS) supplemented with 40 ng/mL M-CSF and then plated in 48 or 96 well plate.

### In vitro TRAP staining

RAW264.7 cells and BMMs were seeded in 48 or 96-well plates. After 24 h, the cells were treated with 50 ng/mL RANKL and different concentrations of ACT001 (0, 0.625, 1.25, 2.5, 5 µM) for 5–6 days. After osteoclast differentiation, the cells were washed with phosphate-buffered saline (PBS), fixed with 4% paraformaldehyde for 15 min, and stained with a TRAP staining kit according to the manufacturer’s instructions. The number of osteoclasts (cells with three or more nuclei) per well was measured using Image J software.

### Actin-ring staining

Actin-ring formation experiments in RAW264.7 cells and BMMs were performed as previous procedure (Tao et al. 2021). The differentiated osteoclasts were fixed with 4% paraformaldehyde for 15 min, permeabilized with 0.1% Triton X-100 for 5 min, and incubated with Alexa Fluor 488-phalloidin for 30 min. The cells were washed with PBS and then treated with 2-(4-Amidinophenyl)-6-indolecarbamidine dihydrochloride (DAPI). Finally, the cells were imaged with fluorescence microscope (Nikon, Tokyo, Japan).

### Cell viability assay

RAW264.7 cells, BMMs and MC3T3-E1 cells were seeded in 96-well plates at indicated density for 24 h. And then the cells were treated with 0, 0.625, 1.25, 2.5, 5, 10, 20 µM ACT001 for 48 h. 10 µL CCK-8 reagent was added into each well and incubated for another 1 h at 37 ^o^C. The absorbance at 450 nm was measured using a microplate reader (Bio Tek, Vermont, USA).

### Bone resorption test

BMMs were cultured in 96-well plates with and without the bone slices, and incubated with 50 ng/mL RANKL for 6 days until the osteoclasts formed, BMMs were subjected to TRAP staining in 96-well plate without bone slices. Then BMMs cultured on the bone slices were treated with 1.25, 2.5 and 5 µM ACT001 and 50 ng/mL RANKL for another 3 days, then remove cells from bone slices by sonicating 5–15 min, and the bone slices were stained with 1% toluidine blue.

### Quantitative RT-PCR analysis

3 × 10^5^/well RAW264.7 cells were seeded in 6-well plates. After 24 h, the cells were treated with 50 ng/mL RANKL and different concentrations of ACT001 or MCC950 for 48 h. BMMs cells were seeded in 48-well plates and were treated with 40 ng/mL M-CSF and 50 ng/mL RANKL in the presence of different concentrations of ACT001 for 48 h. Then total RNA was extracted using the TRIzol reagent (Takara, Japan) according to the manufacturer’s instructions. The quantity of RNA was measured using Nanodrop spectrophotometer (Thermo Fisher Scientific, USA). And the cDNA was reversed-transcribed using a reverse transcription kit (TransGen Biotech, Beijing, China). Then, real-time quantitative PCR was performed using SYBR Green PCR MasterMix (TransGen Biotech, Beijing, China). The following primers used in the PCR reaction: *NFATc1*, forward 5’-GGTGCTGTCTGGCCATAACT-3’, reverse 5’-GAAACGCTGGTACTGGCTTC-3’; *TRAP*, forward 5’-ACGGCTACTTGCGGTT.

TCA-3’, reverse 5’-TCCTTGGAGGCTGGTCTT-3’; *Ctsk*, forward 5’-AGGCGGCTATATGACCACTG-3’, reverse 5’-TCTTCAGGGCTTTCTCGTTC-3’; *Dc-stamp*, forward 5’-TCTGCTGTATCGGCTCATCTC-3’, reverse 5’-ACTCCTTGGGTTCCTTGCTT-3’; *GAPDH*, forward 5’-AACTTTGGCATTGTG.

GAAGG-3’, reverse 5’-ACACATTGGGTAGGAACA-3’.

MC3T3-E1 cells were seeded in 6-well plates. After 24 h, the cells were treated with osteogenic induction medium (OIM, complete α-MEM medium containing 10 nM dexamethasone, 50 µg/mL vitamin C, and 5 mM β- glycerophosphate) (Zhang et al. 2025) and different concentrations of ACT001 for 7 d. and then total RNA was extracted and real-time quantitative PCR was performed. The following primers used in the PCR reaction: *Runx2*, forward 5’-CCAACCGAGTCATTTAAGGCT-3’, reverse 5’-GCTCACGTCGCTCATCTTG-3’; *COL 1*, forward 5’-GCTCCTCTTAGGGGCCACT-3’, reverse 5’- CCACGTCTCACCATTGGGG-3’; *OPN*, forward 5’-AGCAAGAAACTCTTCCAAGCAA-3’, reverse 5’-GTGAGATTCGTCAGATTCATCCG-3’; *OCN* forward 5’-GGGAGACAACAGGG.

AGGAAAC-3’, reverse 5’-CAGGCTTCCTGCAGGTACCT-3’; *GAPDH* forward 5’-AGGTCGGTGTGAACGGATTTG-3’, reverse 5’- GGGGTCGTTGATGGCAA.

CA-3’.

### Western blot analysis

RAW264.7 cells were lysed in lysis buffer with protease and phosphatase inhibitor. And the protein concentrations were quantified with a BCA assay (Thermo Fisher Scientific, Waltham, MA, USA). The normalized samples were separated by SDS-PAGE and transferred to polyvinylidene difluoride (PVDF) membranes. The membranes were blocked with blocking solution and incubated with primary antibodies, followed by incubation with anti-rabbit or anti-mouse horseradish peroxidase-conjugated secondary antibodies. The images were captured with a chemiluminescence imaging system (Biorad, USA). The intensity of bands was calculated by Image J software.

### ALP and ARS staining

A BCIP/NBT ALP Color Development Kit was used for ALP staining according to the manufacturer’s instruction. In brief, MC3T3-E1 cells in plates were washed with PBS three times and then fixed with 4% paraformaldehyde for 15 min. At the end of fixing, cells were rinsed with deionized water for 3 times, followed by incubation of BCIP/NBT solution for 18 h in the dark. Then BCIP/NBT solution was discarded and cells were rinsed with deionized water twice.

To perform ARS staining, cells were fixed in 4% paraformaldehyde for 15 min, and subsequently rinsed with deionized water and stained ARS Solution with gentle agitation. Cells were rinsed three times with deionized water.

### OVX-induced osteoporosis mouse model

All animal experiments were performed according to the institutional animal care guidelines established by the Institutional Animal Care and Use Committee of Hebei University. Female C57/BL6J mice at 7–8 weeks were purchased from Vital River Laboratory Animal Technology (Beijing, China). Mice were randomly divided into 5 groups (*n* = 5 in each group): Sham group, OVX group, OVX + 50 mg/kg ACT001 group (ACT001-L), OVX + 100 mg/kg ACT001 group (ACT001-H) and OVX + 0.9 mg/kg ALN group. For surgically bilateral ovariectomy, the ovaries were removed under anesthesia. And a week after surgery, ACT001 and ALN were administrated by oral gavage once a day for seven weeks. The sham group and OVX group were treated with saline. Finally, the femurs were evaluated using micro-computed tomography (micro-CT) (Bruker, Kontich, Belgium). And the tibias were collected for histological and immunohistochemical analyses.

### Micro-CT scanning and analysis

The left femurs (*n* = 5) were analyzed using the SkyScan 1172 high-resolution micro-CT scanner. The scanning parameters were 50 kV (voltage) and 199 mA (current) and the µCT images were obtained. Then, the processed 3D images were imported into CTAn software (Bruker micro-CT, Kontich, Belgium) to measure and analyse the bone volume/tissue volume (BV/TV), trabecular number (Tb.n), trabecular thickness (Tb.Th), trabecular pattern factor (Tb.pf).

### Histological and immunohistochemical analyses

The left tibias were fixed in 4% paraformaldehyde, then decalcified in ethylenediaminetetraacetic acid (EDTA), embedded in paraffin and sliced into 5 μm thick sections using a microtome. Sections were then subjected to hematoxylin and eosin (H&E), TRAP and immunohistochemical (IHC) staining. The major organ including heart, liver, spleen, lung and kidneys were fixed in 4% paraformaldehyde, embedded in paraffin and sliced into 5 μm thick sections using a microtome. Sections were then subjected to hematoxylin and eosin (H&E) staining. The stained sections were photographed using microscope (Nikon, Tokyo, Japan).

### Serum ELISA assay

The concentration of procollagen type I N-propeptide (PINP), bone alkaline phosphatase (BALP), total TRAP, collagen type I C-telopeptide (CTX-1) in the serum was detected using ELISA kit according to the reagent manufacturer’s instructions. In short, the eyeball blood of the above-mentioned OVX mice was centrifuging at 4 ^o^C for 30 min at 1000 rpm. The supernatant was taken for subsequent experiments. The standard substance and serum to be tested were added to a 96-well plate and incubated for 90 min. Biotinylated detection, HRP conjugate, substrate reagent, and stop solution were successively added. OD value of each well was measured at 450 nm by Multimode Microplate Reader.

### Statistical analysis

All results are presented as the mean ± standard error of the mean (SEM) from at least three independent experiments. Data were analyzed by Student’s t-test or one-way analysis of variance (ANOVA) of comparison of the differences in each parameter using the GraphPad Prism software. P values less than 0.05 were considered statistically significant.

## Results

### ACT001 suppresses RANKL-induced osteoclast differentiation

**Fig. 1:**
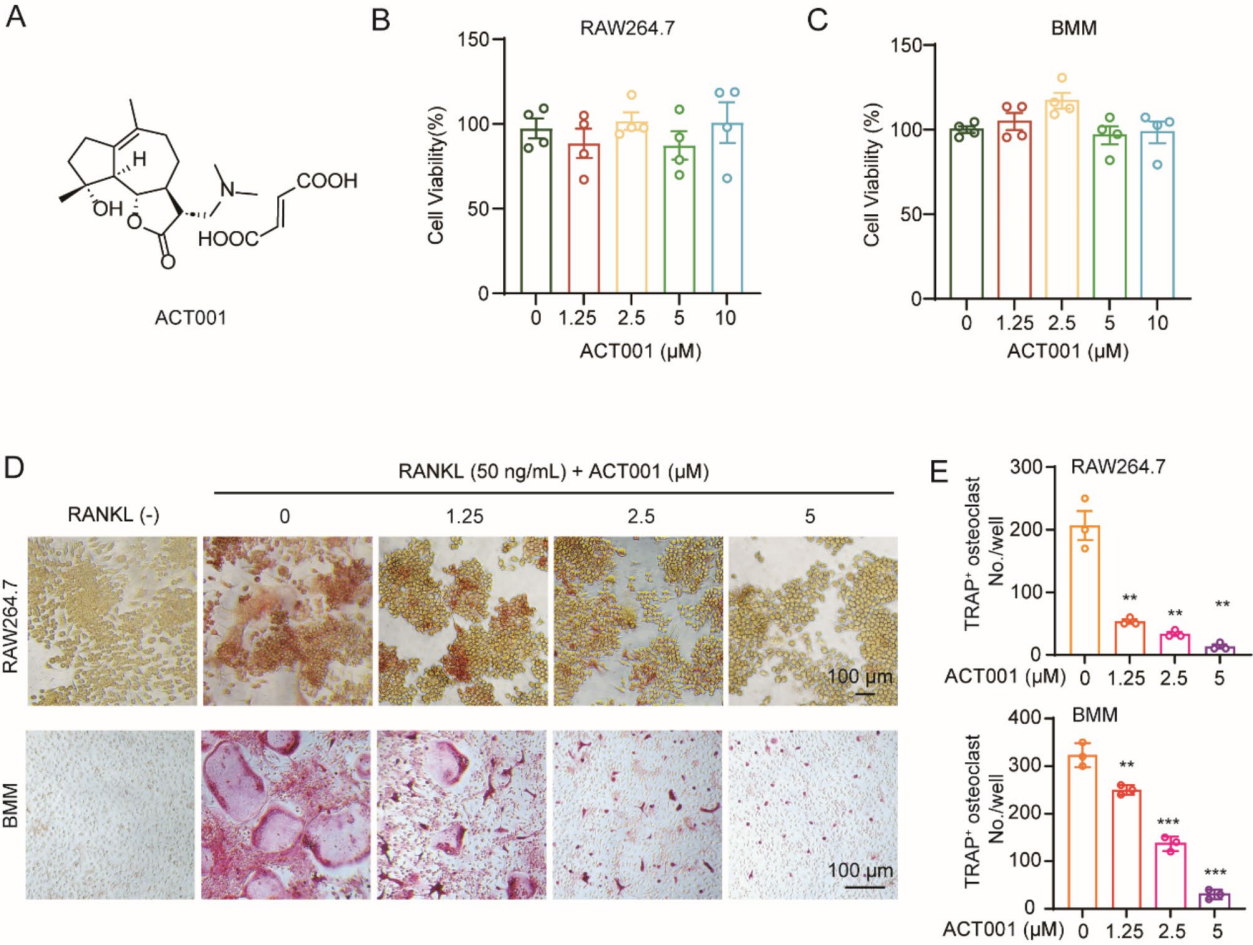
ACT001 suppressed RANKL-induced osteoclast differentiation in vitro. (**A**) The chemical structure of ACT001. (**B**-**C**) RAW264.7 cells (**B**) and BMMs (**C**) were treated with different concentrations of ACT001 for 48 h, cell activity was detected using CCK-8 assays. (**D**) RAW264.7 cells and BMMs were treated with RANKL in the presence of different concentrations of ACT001 for 5–6 days and subjected to TRAP staining, then visualized using a light microscope. Scale bar, 100 μm. (**E**) Quantitative statistics of the TRAP-positive multinucleated cells (nuclei ≥ 3). Data are presented as mean ± SEM. ** *p* < 0.01, *** *p* < 0.001

To investigate the role of ACT001 (Fig. 1A) on osteoclast differentiation, RAW264.7 and BMMs cells were used to induce osteoclast formation. Firstly, we evaluated the cell viability of ACT001 against RAW264.7 cells and BMMs, and the result of CCK-8 showed that there was no obvious toxicity of ACT001 to the cells at the maximum concentration of 10 µM within 48 h (Fig. 1B-C). Then, the RAW264.7 cells and BMMs were incubated with 50 ng/mL RANKL and different concentrations of ACT001 for 5–6 days and stained with TRAP respectively. As shown in Fig. 1D and E, the number of multi-nuclear TRAP^+^ osteoclasts significantly increased after RANKL stimulation in both RAW264.7 cells and BMMs. Compared with RANKL group, ACT001 could efficiently inhibit the osteoclastogenesis in a dose-dependent manner. To further validate the inhibitory effect of ACT001 for osteoclast formation during different stages, 5 µM ACT001 was added to RAW264.7 cells with 50 ng/mL RANKL at four indicated time points. Notably, ACT001 significantly reduced the numbers of TRAP^+^ cells at each stage of osteoclastogenesis, especially during the early stage (day 1–2) (Fig. S1).

### ACT001 inhibits RANKL-induced F-actin ring formation and osteoclast resorptive function

**Fig. 2:**
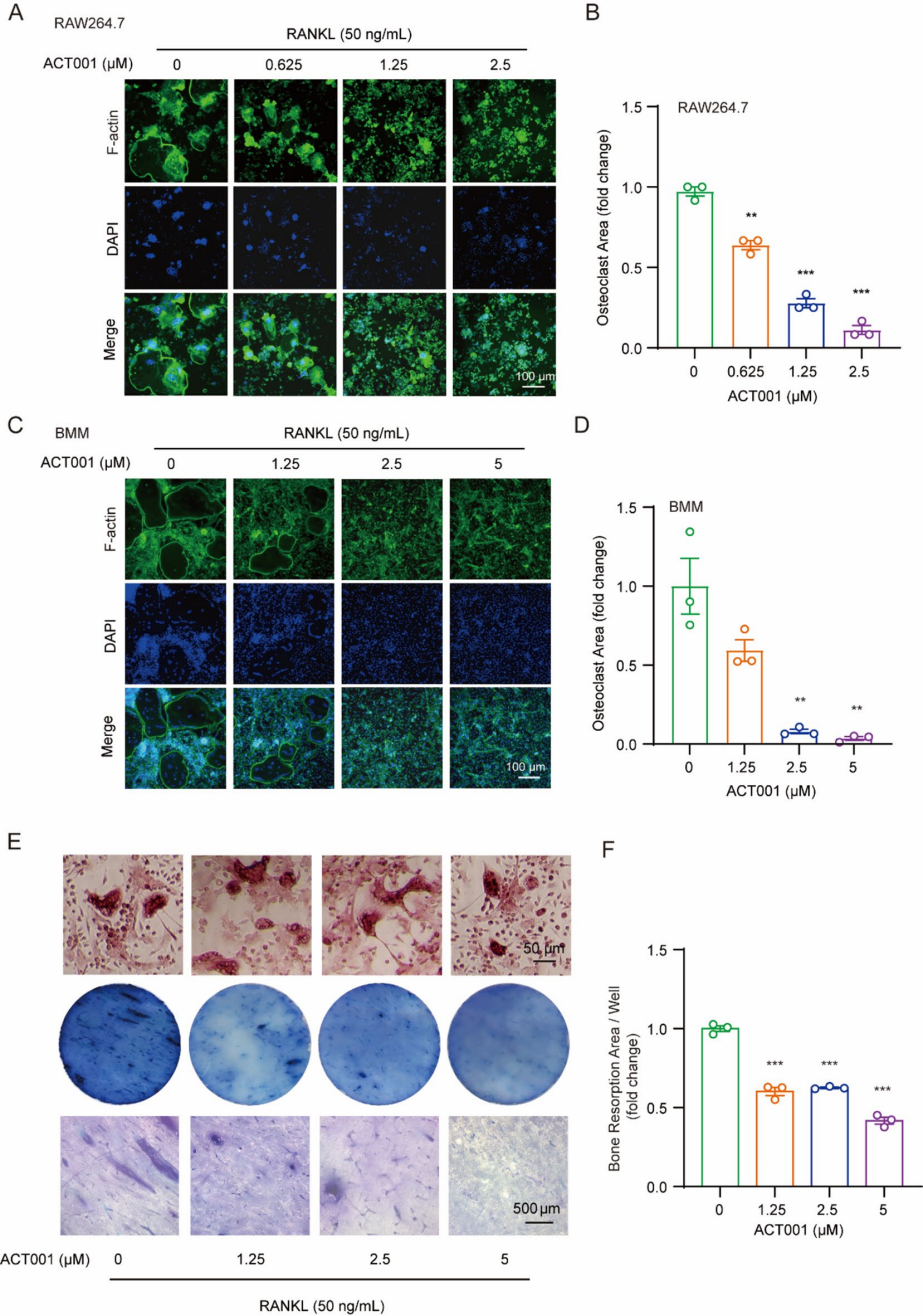
ACT001 inhibited the F-actin ring formation and osteoclast resorptive function. (**A**, **C**) RAW264.7 cells (**A**) and BMMs (**C**) were treated with RANKL in the presence of different concentrations of ACT001 for 5–6 days. The cells were stained with Alexa Fluor 488-phalloidin and DAPI, and then imaged with fluorescence microscope. Scale bar, 100 μm. (**B**, **D**) Quantitative statistics of the osteoclast area in each visual field. (**E**) BMMs were cultured in 96-well plates with and without the bone slices, and incubated with 50 ng/mL RANKL for 6 days until the osteoclasts formed, BMMs were subjected to TRAP staining in 96-well plate without bone slices. Then BMMs cultured on the bone slices were treated with ACT001 and RANKL for another 3 days, and the bone slices were stained with toluidine blue. (**F**) Quantification of the resorption pit area per bone slice. Data are presented as mean ± SEM. ** *p* < 0.01, *** *p* < 0.001

The formation of filamentous actin (F-actin) rings plays an important role in osteoclast differentiation. Herein, we evaluated the inhibitory effects of ACT001 on the F-actin ring formation through co-staining with the F-actin and DAPI in RAW264.7 cells. With the increasing concentration of ACT001, the number and size of F-actin rings induced by RANKL were both significantly decreased (Fig. 2A-B). Next, we tested the osteoclast resorptive activity in the presence of ACT001 using bone resorption pit assay. BMMs were cultured in 96-well plates with and without the bone slices, and incubated with 50 ng/mL RANKL for 6 days. As shown in Fig. 2E (the upper panel), the TRAP staining results showed that multinucleated osteoclasts have formed. After that, the BMMs cultured on the bone slices were treated with ACT001 and RANKL for another 3 days, then the cells were removed from bone slices, and the bone slices were stained with toluidine blue. As shown in Fig. 2E (the lower panel) and 2 F, the resorption pit area was dramatically reduced with the increase of ACT001 concentration.

### ACT001 down-regulates the expression of osteoclast-related genes and proteins

**Fig. 3:**
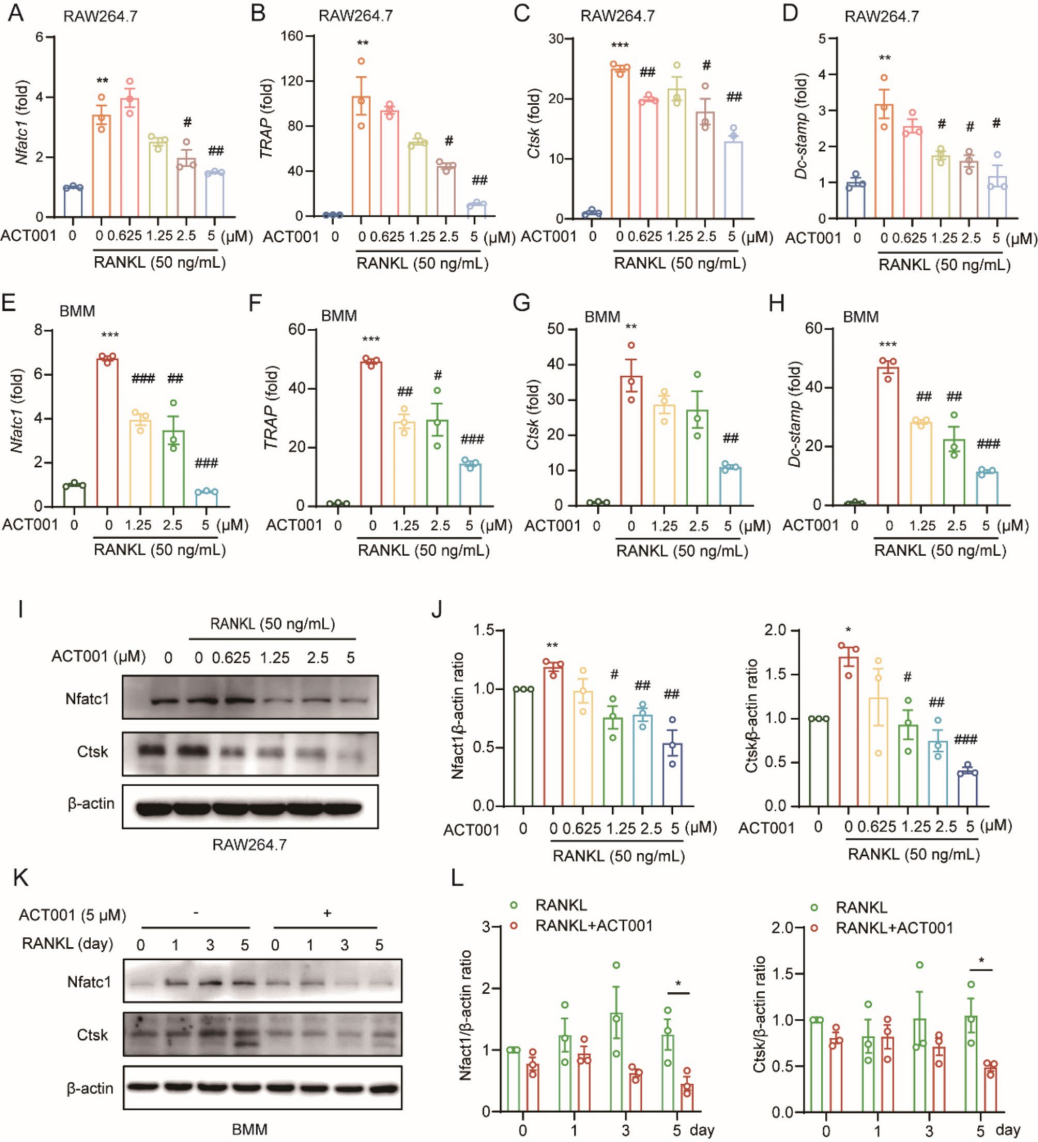
ACT001 restrained RANKL-induced osteoclast-related genes and proteins. (**A**-**D**) RAW264.7 cells were treated with RANKL in the presence of different concentrations of ACT001 for 2 days, and the relative expression of osteoclast-related genes *Nfatc1*(**A**), *TRAP* (**B**), *Ctsk* (**C**) and *Dc-stamp* (**D**) was measured via real-time PCR. (**E**-**H**) BMMs were treated with RANKL in the presence of different concentrations of ACT001 for 2 days, and the relative expression of osteoclast-related genes *Nfatc1*(**E**), *TRAP* (**F**), *Ctsk* (**G**) and *Dc-stamp* (**H**) was measured via real-time PCR. (**I**) RAW264.7 cells were treated with RANKL in the presence of different concentrations of ACT001 for 3 days, the protein expression levels of Nfatc1 and Ctsk were detected via western blot. (**J**) The relative ratio of the gray band value of Nfatc1 and Ctsk to that of β-actin. (**K**) RAW264.7 cells were induced by RANKL and treated with 5 µM ACT001 at different time point, the protein expression levels of Nfatc1 and Ctsk were detected via western blot. (**L**) The relative ratio of the gray band value of Nfatc1 and Ctsk to that of β-actin. Data are presented as mean ± SEM. * *p* < 0.05, ** *p* < 0.01, *** *p* < 0.001 versus vehicle group; ^#^*p* < 0.05, ^##^*p* < 0.01, ^###^*p* < 0.001 versus RANKL-treated group

Further, we verified the impact of ACT001 on the expression of osteoclast-related genes and proteins. RAW264.7 cells and BMMs were treated with RANKL and different concentrations of ACT001 for 48 h, osteoclast related genes including *Nfatc1*, *TRAP*, Cathepsin K (*Ctsk*), dendritic cell-specific transmembrane protein (*Dc-stamp*) were tested by qRT-PCR. And the results showed that the mRNA expressions of *Nfatc1*, *TRAP*, *Ctsk* and *Dc-stamp* were significantly up-regulated in the RANKL-induced group in both RAW264.7 cells (Fig. 3A-D) and BMMs (Fig. 3E-H), but ACT001 down-regulated the expressions of these genes in a dose-dependent manner. Consistently, RANKL-induced upregulation of Nfatc1 and Ctsk protein expressions were significantly inhibited by ACT001 at concentration of 1.25, 2.5 and 5 µM after treatment for 48 h in RAW 264.7 cells (Fig. 3I-J). Meanwhile, Immunofluorescence results showed that ACT001 could significantly inhibit the expression of Nfact1 in the cytoplasm (Fig. S2). Moreover, we also treated RAW264.7 cells with RANKL or RANKL + ACT001 (5 µM) for 1 d, 3 d and 5 d, respectively. As shown in Fig. 3K-L, ACT001 continuously inhibited the expression of Nfatc1 and Ctsk. These results above suggested that ACT001 could significantly suppress the differentiation and resorption of osteoclasts through inhibiting the Nfatc1 activation.

### ACT001 mainly inhibits RANKL-induced activation of the NF-κB signaling pathway

**Fig. 4:**
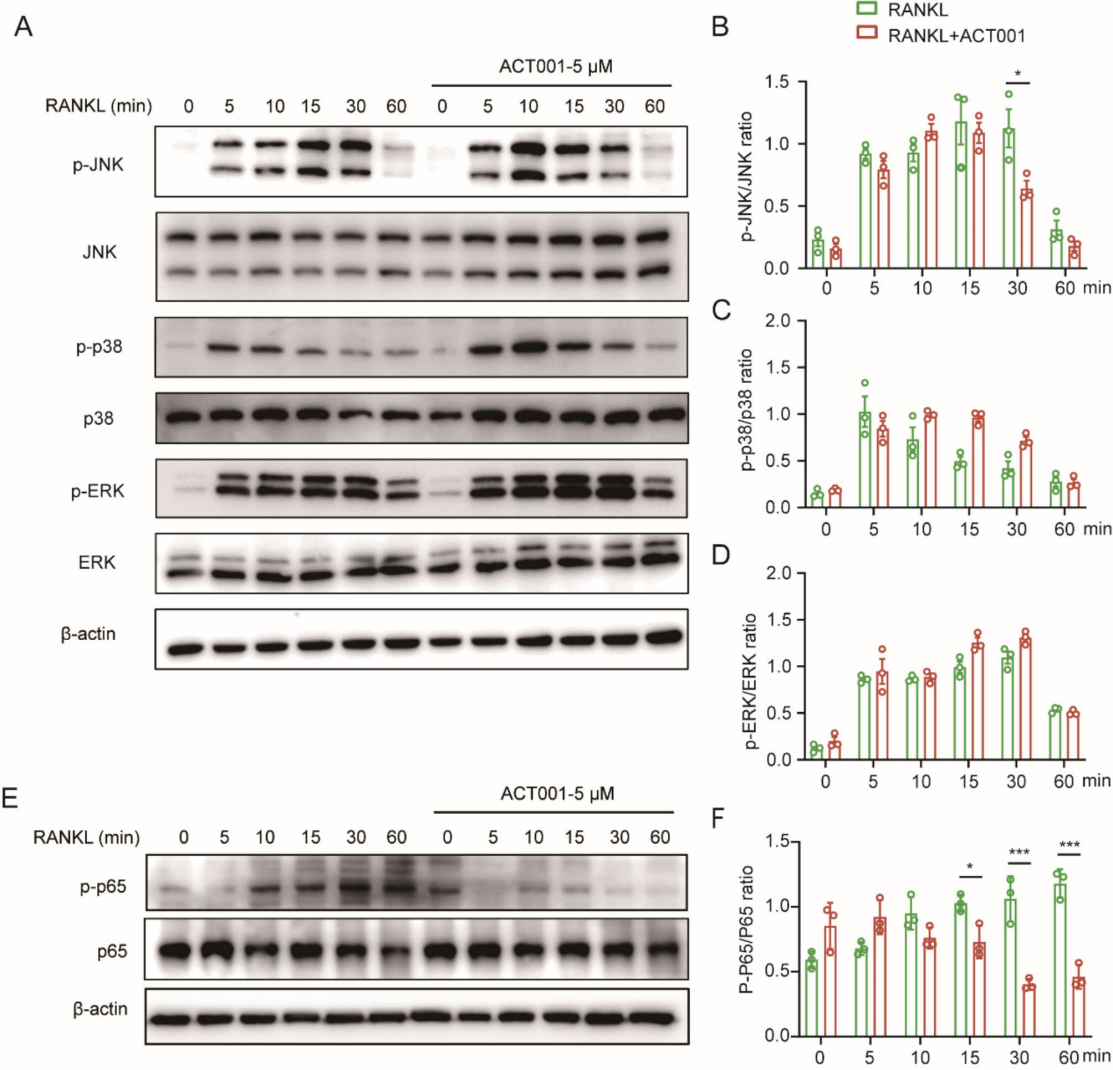
ACT001 inhibited RANKL-induced activation of NF-κB signaling. RAW264.7 cells were pretreated with or without 5 µM ACT001 for 2 h, then incubated with 50 ng/mL RANKL for different times. (**A**) The protein expression levels of MAPK pathway were detected via western blot. (**B**-**D**) The relative ratio of phosphorylated bands of JNK (**B**), P38 (**C**), and ERK (**D**) to the gray values of total protein bands. **(E) **The protein expression levels of NF-κB pathway were detected via western blot. (**F**) The relative ratio of phosphorylated bands of p65 to the gray values of total protein bands. Data are presented as mean ± SEM. * *p* < 0.05, ** *p* < 0.01

To explore the possible mechanism of ACT001, we investigated the related proteins in MAPKs and NF-κB pathways, which are two primary signals involved in osteoclast differentiation. RAW264.7 cells were pretreated with or without 5 µM ACT001 for 2 h, then incubated with 50 ng/mL RANKL for different times. As shown in Fig. 4A-D, the expression levels of the phospho-JNK (p-JNK), phospho-p38 (p-p38) and phospho-ERK (p-ERK) induced by RANKL were significantly increased in the time-course experiment, and ACT001 treatment decreased the expression level of p-JNK at 30 min induction, but did not decrease the expression of p-p38 and p-ERK. Meanwhile, ACT001 significantly inhibited the phosphorylation of p65 at 15, 30 and 60 min of RANKL induction (Fig. 4E-F). Furtherly, to verify whether ACT001 inhibits NF-κB pathway via influencing the interaction between TLR4 and MD2, the MD2 inhibitor MD2-IN-1 was used to pretreated RAW264.7 cells. As shown in Fig. S3, MD2-IN-1 could not inhibit RANKL-induced the phosphorylation of p65, compared with MD2-IN-1 treated group, but ACT001 and MD2-IN-1 co-treated could reverse the inhibitory effect of ACT001 on RANKL-induced the phosphorylation of p65, indicating that the inhibitory effect of ACT001 may depend on MD2. Together, these results indicated that ACT001 mainly inhibit RANKL-induced the activation of NF-κB pathway.

### ACT001 attenuates RANKL-induced NLRP3 inflammasome activation in RAW264.7 cells

**Fig. 5:**
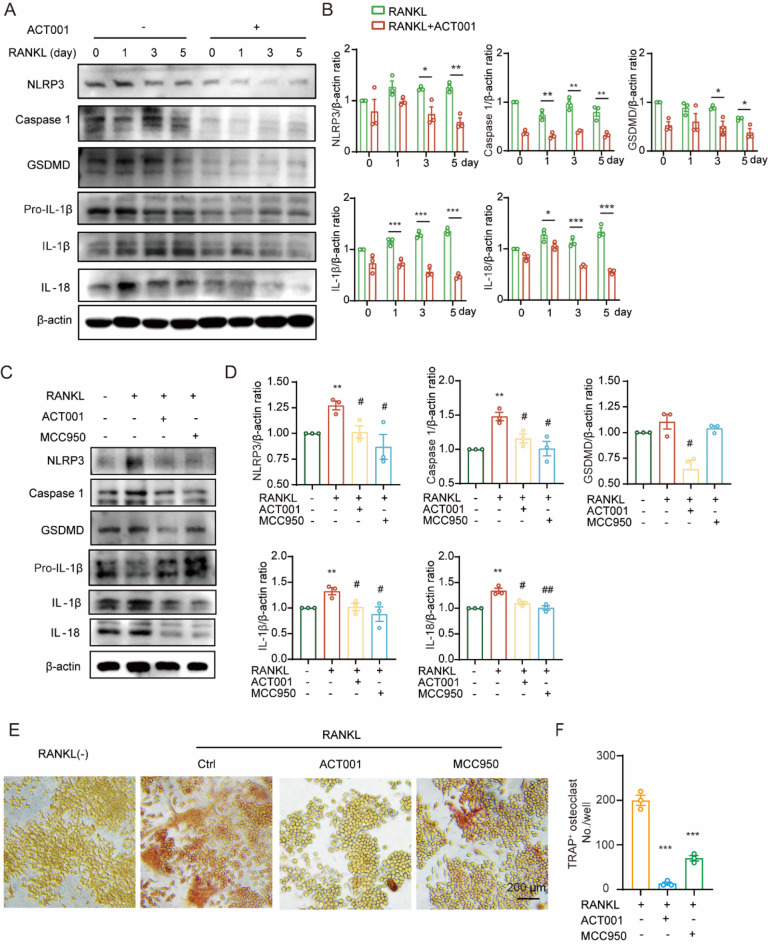
ACT001 inhibited RANKL-induced NLRP3 inflammasome activation in vitro. (**A**) RAW264.7 cells were induced by RANKL and treated with 5 µM ACT001 at different time point, the protein expression levels of NLRP3, Caspase 1, GSDMD, IL-1β and IL-18 were detected via western blot. (**B**) The relative ratio of the gray band value of the NLRP3 cascade proteins (**A**) to that of β-actin. * *p* < 0.05, ** *p* < 0.01, *** *p* < 0.001 versus RANKL group. (**C**) RAW264.7 cells were induced by RANKL and treated with 5 µM ACT001 or 5 nM MCC950 for 48 h, the protein expression levels of NLRP3, Caspase 1, GSDMD, IL-1β and IL-18 were detected via western blot. (**D**) The relative ratio of the gray band value of the NLRP3 cascade proteins (**C**) to that of β-actin. ** *p* < 0.01 versus vehicle group, ^#^*p* < 0.05, ^##^*p* < 0.01 versus RANKL-treated group. (**E**) RAW264.7 cells were induced by RANKL and treated with 5 µM ACT001 or 5 nM MCC950 for 5 days and subjected to TRAP staining, then visualized using a light microscope. Scale bar, 200 μm. (**F**) Quantitative statistics of the TRAP-positive multinucleated cells (nuclei ≥ 3). *** *p* < 0.001 versus RANKL-treated group. Data are presented as mean ± SEM

NLRP3 inflammasome is a multiprotein complex that mediates inflammation and induces pyroptosis through activating caspase-1, gasdermin D (GSDMD), interleukin (IL)-1β, IL-18, and its activation is involved in osteoclast differentiation and bone resorption (Jiang et al. 2021). Herein, in order to assess the inhibitory effects of ACT001 on RANKL-induced NLRP3 inflammasome activation, the RAW264.7 cells were treated with RANKL or RANKL + ACT001 (5 µM) for 1 d, 3 d and 5 d, respectively, and the expressions of NLRP3 inflammasome-related proteins were determined using western blot. Notably, ACT001 significantly suppressed the level of NLRP3, caspase-1, GSDMD, and IL-1β and IL-18 compared to the RANKL-induced group at 3 d and 5 d (Fig. 5A-B). Meanwhile, we also selected MCC950 (a specific and potent inhibitor of NLRP3 inflammasome) as a positive control to compare its effects on NLRP3 activation and osteoclast differentiation with ACT001. As shown in Fig. 5C-D, both ACT001 and MCC950 similarly suppressed the NLRP3 inflammasome activation at indicated concentration. Meanwhile, the mRNA and protein levels of Nfatc1 and Ctsk were reduced after treatment with both of ACT001 and MCC950 in RAW264.7 cells (Fig. S4A-D). In addition, TRAP staining results showed that MCC950 treatment also suppressed the osteoclast formation consistent with ACT001 (Fig. 5E-F). All these results revealed that ACT001 inhibits NLRP3 inflammasome activation in osteoclast.

#### ACT001 does not promote osteoblast differentiation and mineralization in vitro

**Fig. 6:**
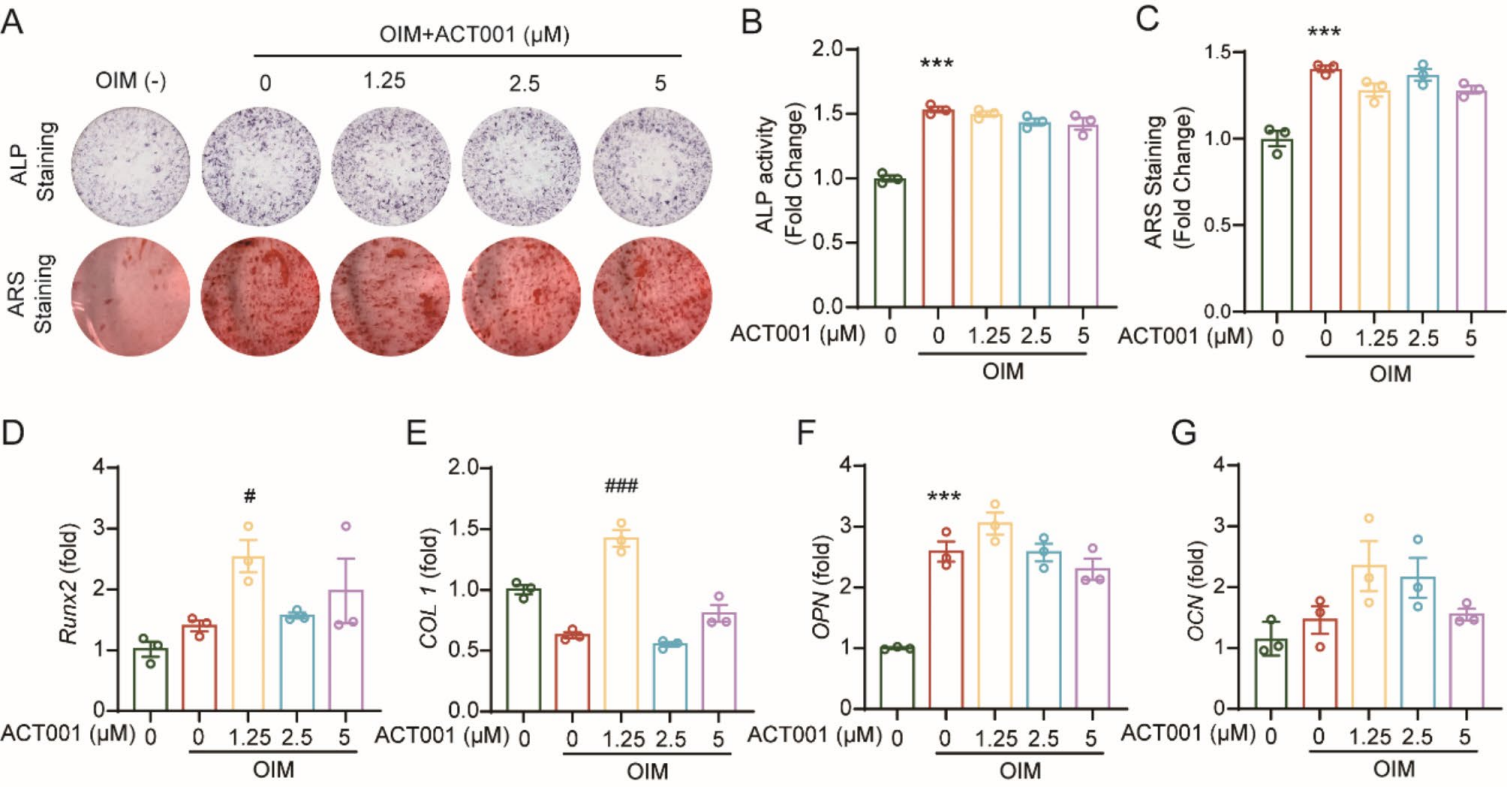
Effect of ACT001 on osteoblast differentiation and mineralization. (**A**) MC3T3-E1 cells were incubated with OIM and different concentrations of ACT001 for 7 days for ALP staining, and incubated with OIM and different concentrations of ACT001 for 21 days for ARS staining, respectively. (**B**) Quantification of the ALP staining areas per plate. (**C**) The ARS bound to MC3T3-E1 cells were dissolved and the released ARS was measured. (**D**-**G**) MC3T3-E1 cells were incubated with OIM and different concentrations of ACT001 for 7 days, and the relative expression of osteoblast-related genes Runx2 (**D**), COL 1 (**E**), OPN (**F**) and OCN (**G**) was measured via real-time PCR. Data are presented as mean ± SEM. *** *p* < 0.001 versus vehicle group; ^#^*p* < 0.05, ^###^*p* < 0.001 versus OIM-treated group

The effect of ACT001 on osteoblast differentiation and mineralization was further evaluated. Firstly, the cytotoxicity of ACT001 on MC3T3-E1 cells was measured using CCK8 assay, and the result showed that ACT001 did not affect the cell viability at the concentrations tested (Fig.S5). MC3T3-E1 cells were cultured with osteogenic induced medium (OIM) and different concentrations of ACT001 for 7 days and 21 days, and then stained with ALP and ARS respectively. However, compared with OIM-treated group, ACT001 had no effect on the osteoblast differentiation and mineralization (Fig. 6A-C). Meanwhlie, the regulation of mRNA expression of osteoblastgenesis related genes by ACT001 was detected following 7 days with OIM. As shown in Fig. 6D-G, compared with OIM-treated group, ACT001 significantly up-regulated the mRNA expression of runt-related transcription factor 2 (RUNX2) and type I collagen (COL 1) at 1.25 µM, but no obvious changes of the mRNA expression of RUNX2, COL 1, osteopontin (OPN), and osteocalcin (OCN) were observed at 2.5 and 5 µM concentration. Taken together, these results indicate that ACT001 does not promote osteoblast differentiation and mineralization in vitro.

### ACT001 prevents bone loss in OVX mouse models

**Fig. 7:**
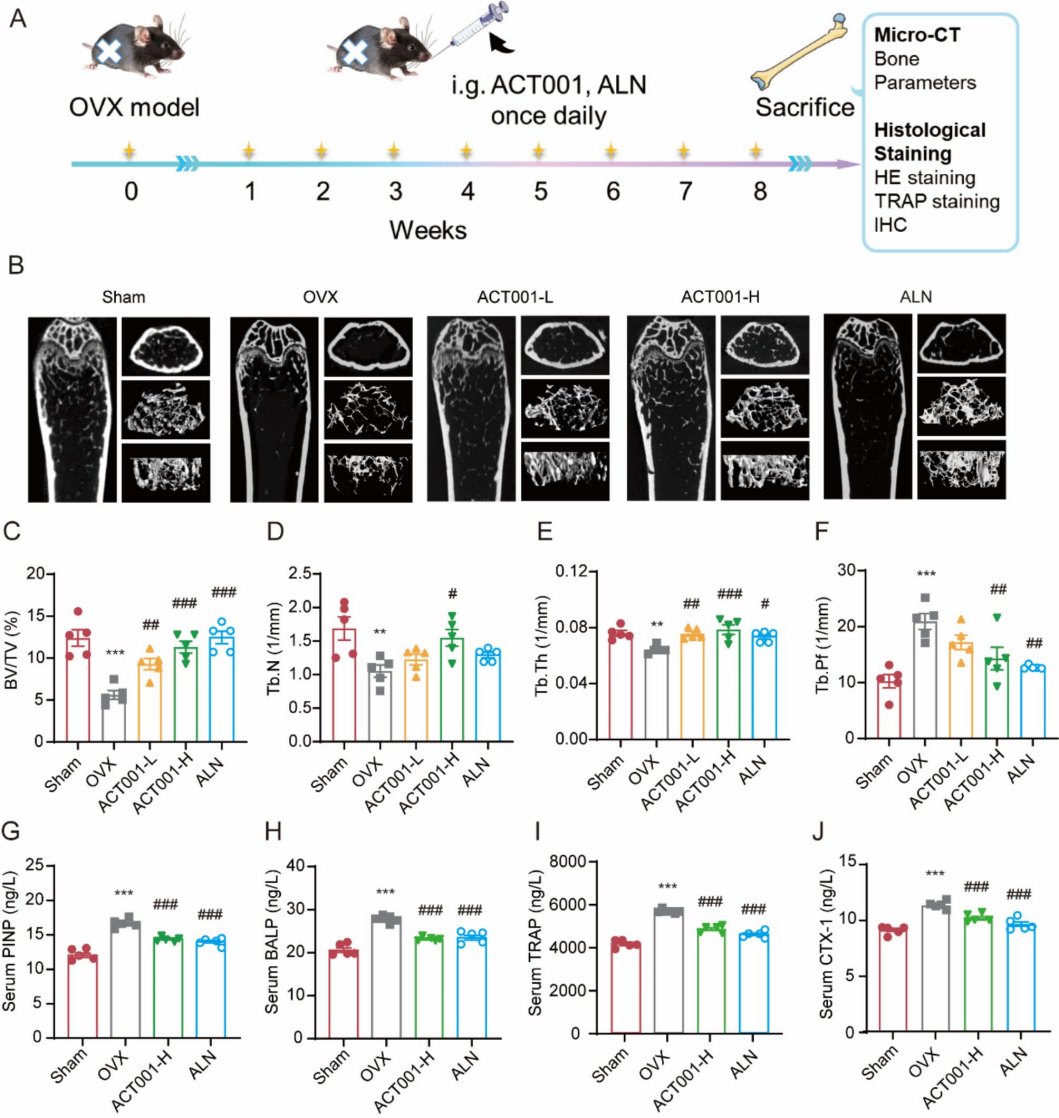
ACT001 inhibited OVX-induced bone loss. (**A**) Schematic diagram of the in vivo experiments. (**B**) Representative micro-CT images of different sections of mouse femurs. (**C**-**F**) Quantitative analyses of bone structural parameters, including BV/TV, Tb.N, Tb.Th, Tb.Pf. (**G**-**H**) The levels of the bone formation marker PINP (**G**) and BALP (**H**) in the mice serum in indicated groups. (**I**-**J**) The levels of the bone resorption marker TRAP (**I**) and CTX-1 (**J**) in the mice serum in indicated groups. Data are presented as mean ± SEM. ** *p* < 0.01, *** *p* < 0.001 versus sham group, ^#^*p* < 0.05, ^##^*p* < 0.01, ^###^*p* < 0.001 versus OVX group

To further investigate the in vivo protective effect of ACT001 on osteoporosis, the classical OVX-induced osteoporosis mouse models were established. Herein, we used alendronate (ALN, Fisamax), a commonly prescribed anti-resorptive medication as a positive control. One week after surgery, the sham group was treated with saline, the OVX mice were randomly divided into four groups and orally administrated with saline, ACT001 (50 mg/kg or 100 mg/kg) and ALN (0.9 mg/kg) daily for seven weeks (Fig. 7A). As illustrated in Fig. 7B, the three-dimensional (3D) reconstruction of the femurs showed that both ACT001 and ALN reduced the extensive trabecular bone loss compared with that in the OVX group. The bone structural parameters in each group were analyzed by micro-CT software to further quantify these changes. Compared with the sham group, the OVX group showed significantly lower BV/TV, Tb.N, and Tb.Th values, and a significantly higher Tb.Pf value (Fig. 7C-F). However, oral administration of ALN and ACT001 increased the values of BV/TV, Tb.N and Tb.Th, and decreased the Tb.Pf values compared with the OVX group. And no significant difference was observed between the ACT001-H group and ALN group. Additionally, we further evaluated the mice serum bone metabolism markers in sham, OVX, ACT001-H, and ALN group. As shown in Fig. 7G-J, compared with sham group, the bone formation marker PINP, BALP and bone resorption marker TRAP, CTX-1 were all significantly increased to different degrees in the OVX group, indicating that the mice bone conversion rate in the OVX group was increased. While both ACT001-H and ALN significantly down-regulated serum bone conversion indices, and the inhibitory effect was comparable, indicating that the bone tissue reconstruction became stable.

**Fig. 8:**
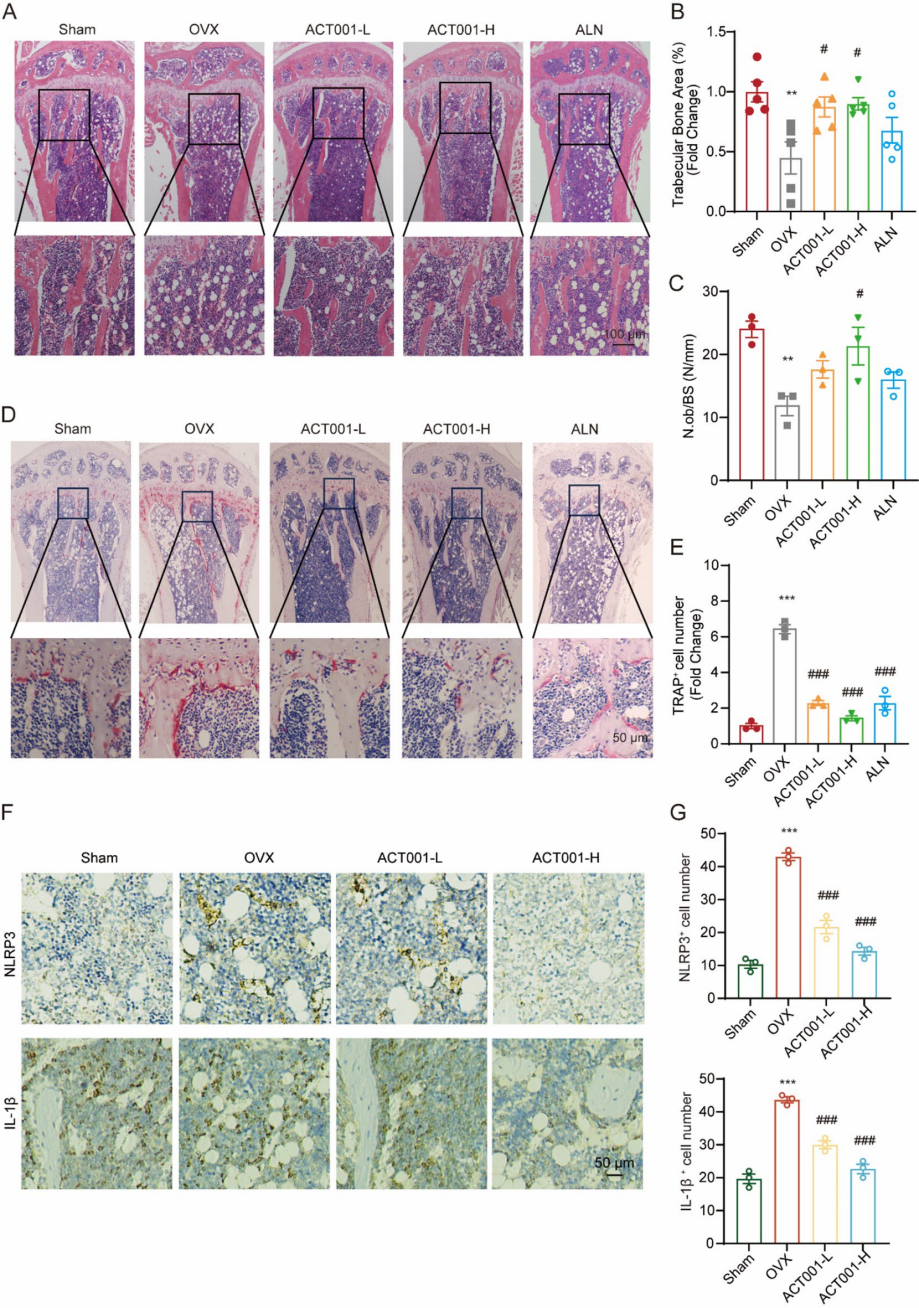
ACT001 reduced osteoclast number and the expression of NLRP3 and IL-1β in vivo. (**A**) Representative H&E staining in tibia bone sections of the sham, OVX, ACT001-L, ACT001-H and ALN group. Scale bar, 100 μm. (**B**) Quantitative analysis of bone trabecular area in tibia bone sections stained with H&E. (**C**) Quantitative analyses of histomorphometric bone parameters of N.Ob/BS (N/mm). (**D**) Representative TRAP staining in tibia bone sections of the sham, OVX, ACT001-L, ACT001-H and ALN group. Scale bar, 50 μm. (**E**) Quantitative analysis of the numbers of TRAP positive cell in indicated groups. (**F**) Representative IHC staining for NLRP3 and IL-1β. Scale bar, 200 μm. (**G**) Quantitative analysis of NLRP3 and IL-1β positive cells in the bone sections from the sham, OVX, ACT001-L and ACT001-H group. Data are presented as mean ± SEM. ** *p* < 0.01, *** *p* < 0.001 versus sham group, ^#^*p* < 0.05, ^###^*p* < 0.001 versus OVX group

Subsequently, H&E and TRAP staining were carried out to evaluate the histological changes of the tibias in each group. As shown in Fig. 8A-C, compared with the Sham group, there was an obvious bone trabeculae loss, and the number of osteoblasts (N.Ob/BS) was also significantly decreased in the OVX group, while ACT001-H and ALN treatment preserved the bone trabeculae structure, N.Ob/BS was significantly increased in the ACT001-H group. TRAP staining showed that the number of osteoclasts around the bone trabeculae was significantly increased in the OVX group, but obviously reduced after treatment with ACT001 and ALN (Fig. 8D-E). Also, the expressions of NLRP3 and IL-1β in tibias were detected using IHC analysis. The results showed that the amount of NLRP3- and IL-1β- positive cells in OVX group were higher than that in the Sham group, while ACT001 treatment significantly decreased the expression of NLRP3 and IL-1β (Fig. 8F-G). Therefore, our study indicated that ACT001 can protect against OVX-induced bone loss, showing the therapeutic potential for osteoporosis.

To investigate the biosafety of ACT001 in vivo, we collected the main organs (i.e., heart, liver, spleen, lung and kidneys) in each group for histopathological examination. As shown in Fig.S6, the H&E staining showed that lipid droplets were drastically increased in the liver of the OVX group compared to the sham group, and this may be caused by the disturbance of lipid metabolism in the liver induced by estrogen deficiency, and ACT001 or ALN treatment markedly lowered lipid accumulation in the liver. In addition to that, there was no obvious signs of inflammation and abnormality in histopathology was found, further indicating that ACT001 is safe for the in vivo treatment of OP.

## Discussion

Osteoporosis is a common chronic metabolic bone disease affecting bone health and overall quality of life in older adults, and it is known as “the silent killer of the 21st century” and becomes a major public health concern worldwide (Aibar-Almazan et al. 2022). It has been caused a huge financial burden to the world with the increasing incidence of the OP (Shen et al. 2022; Rashki Kemmak et al. 2020). Therefore, the prevention and management of OP has become an important healthcare challenge. Currently, there are several drugs available for treating OP, including calcium and vitamin D supplementation, hormonal agents, antiresorptive agents, and so on (Barnsley et al. 2021). However, the adverse effects and poor conpliance of the anti-OP drugs reduce the effectiveness of the OP treatment (Hesari et al. 2023). Therefore, developing novel clinical drugs for OP prevention is imperative.

Given the complex and multifactorial pathophysiological features, the main goal of OP treatment is to maintain bone homeostasis and reduce the risk of fracture (Muniyasamy and Manjubala 2024). The bone homeostasis is primarily regulated by the activity levels of osteoblasts and osteoclasts together (Zhivodernikov et al. 2023). In our study, we also confirmed that ACT001 has no significant effect on the differentiation or mineralization nodules in MC3T3-E1 cells. Therefore, we focused on the inhibitory effects of ACT001 on the osteoclast activities.

During osteoclast formation and differentiation, RANKL binding to the RANK expressed by osteoclast precursors leads to the activation of signaling cascades, including NF-κB and MAPK pathways (Lee et al. 2018), ultimately resulting in the activation of Nfatc1 (Maeda et al. 2022). In this study, we investigated the biological effects of ACT001 on osteoclast formation and differentiation in vitro and OVX-induced OP in vivo. Our results indicate that ACT001 can efficiently inhibit RANKL-induced osteoclast formation by suppressing the NF-κB signaling pathway activation, and thereby inhibiting the transcriptional activation of Nfatc1.

Previous studies have suggested estrogen deficiency is the main cause that brokens the balance of bone metabolism, and the estrogen was used to treat OP mainly through four effector cells such as osteoblasts, osteoclasts, osteocytes and T cells (Khosla et al. 2012). However, besides the effects of hormones on bone metabolism, chronic inflammation has been reported to promote bone loss and the onset of OP (Redlich and Smolen 2012). And chronic inflammatory conditions induced by aging and estrogen deficiency activate NLRP3 inflammasome, which not only accelerates bone resorption, but also inhibits bone formation, thus increasing the risk of OP. The NF-κB signaling pathway is a key mediator of the pro-inflammatory response, and plays a central role in the aggregation of NLRP3 components and the formation of NLRP3 inflammasome activation (An et al. 2019; Afonina et al. 2017), and then promote the activation of pro-Caspase-1. Active Caspase-1 cleaves the pro-IL-18, pro-IL-1β and GSDMD, culminating in the maturation and release of these molecules and inducing the inflammation and pyroptosis (Wang et al. 2020; Liang et al. 2024). Moreover, using an effective NLRP3 inhibitor MCC950 can prevent bone loss in OVX mice (Tao et al. 2021) and diabetic osteoporosis mice (Cai et al. 2024) in vivo. In addition, Urolithin A, an active metabolite from gut microbiota, has reported to suppress RANKL-induced osteoclastogenesis and postmenopausal OP through inhibiting inflammation and downstream NF-κB activated pyroptosis pathway (Tao et al. 2021). Alloferon-1 could also prevent OVX-induced OP through dampening the NLRP3/capsae-1/IL-1β/IL-18 signaling pathway (Qiao et al. 2023). All these findings suggest that NLRP3 could be a therapeutic target for human OP.

In this study, we detected the effects of ACT001 and MCC950 on NLRP3-mediated pyroptosis related proteins expression and osteoclast differentiation in vitro. The results showed that both ACT001 and MCC950 significantly decreased the expression of NLRP3, GSDMD, Caspase-1, IL-1β, IL-18 induced by RANKL. Meanwhile, ACT001 showed comparable to or stronger inhibition of osteoclast differentiation than MCC950, indicating that ACT001 may have a broader application prospect in OP therapy. MCC950 is a highly specific potent small-molecule NLRP3 inhibitor, and has shown good efficacy in treatment of autoimmune diseases, cardiovascular diseases, metabolic diseases and other diseases (Li et al. 2022). And clinical studies of MCC950 have also been conducted, but increased serum liver enzyme levels were found in phase II clinical trial for RA, indicating liver toxicity (Mangan et al. 2018). Different from MCC950, in addition to directly interacting with NLRP3 (Luo et al. 2023), ACT001 can also inhibit NF-κB activation and phosphorylation by directly binding IKKβ (Liu et al. 2020; Cai et al. 2022; Li et al. 2020), thereby reducing inflammation response.

ACT001, a prodrug of micheliolide (MCL), is synthesized from the natural product pathenolide (PTL). It has been previously reported that PTL can inhibit RANKL-induced osteoclast differentiation and bone resorbing activity by down-regulating of Nfatc1 induction and c-fos stability (Kim et al. 2014). Moreover, PTL has no direct effects on the cell viability and osteoblastic activities of human periosteum-derived cells (hPDCs) under in vitro cell culture conditions (Park et al. 2020). MCL can also inhibit osteoclast differentiation through inhibiting p38 signaling pathway (Gan et al. 2023). In addition, epoxymicheliolide (EMCL) can inhibit osteoclastogensis and resist OVX-induced OP by suppressing ERK1/2 and Nfatc1 signaling (Long et al. 2022). Taken together, ACT001 and its active metabolites have shown potential anti-OP activities. Moreover, compared to the above compounds, ACT001 is an oral therapeutic agent with better stability, solubility, low toxicity, and minimal side effects, and has been extensively validated in multiple clinical trials (Zhang et al. 2012). In our previous study and clinical trials, ACT001 has shown significant activities against multiple solid tumors, all these findings suggest that ACT001 can be used for the postoperative treatment of ovarian cancer, while preventing the development of estrogen deficiency induced osteoporosis in the future clinical trials. In this study, we only selected the most commonly used OVX mouse model to verify that ACT001 has a good anti-OP effect at a dose of 100 mg/kg, and the therapeutic effects of higher dose of ACT001 on OVX model or other types of OP animal models remain to be elucidated.

## Conclusions

**Fig. 9:**
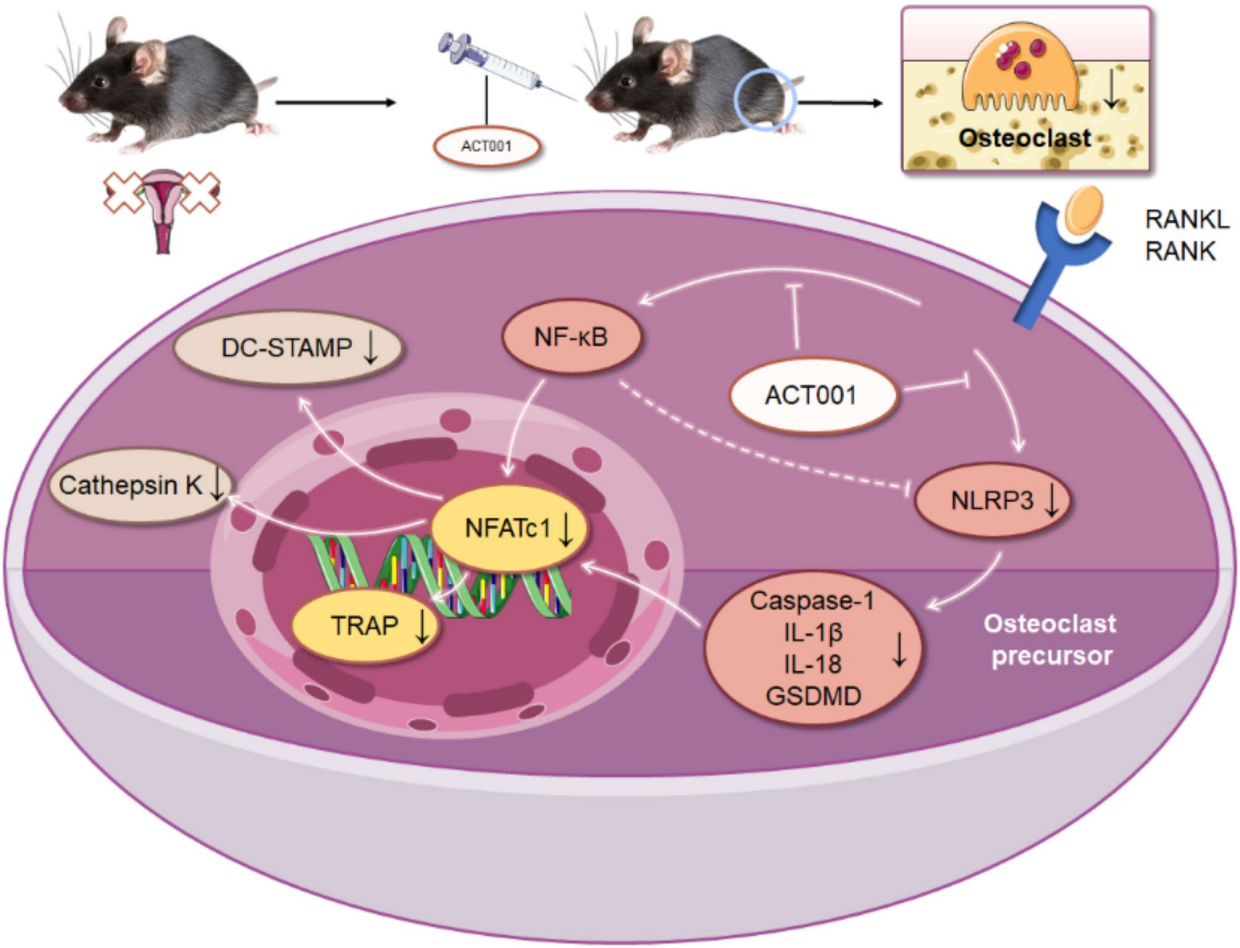
The proposed mechanism of ACT001 inhibition of osteoclastogensis and differentiation via suppressing of NF-κB/NLRP3 pathway and NFATc1 expression

In conclusion, our study shows that ACT001 can inhibit RANKL-induced osteoclastogenesis and differentiation in vitro and attenuate OVX-induced OP in vivo through suppressing NF-κB/NLRP3 pathway and NLRP3-inflammasome-triggered pyroptosis cascades, and inhibiting the transcriptional activation of Nfatc1 (Fig. 9). As an orphan drug by FDA-approved, ACT001 is a novel promising drug for osteoporosis in preclinical and clinical studies.

## Electronic supplementary material

Below is the link to the electronic supplementary material.

Supplementary Material 1

## Data Availability

No datasets were generated or analysed during the current study.

## Abbreviations

OP: Osteoporosis
OVX: Ovariectomy
RANKL: Receptor activator of nuclear factor kappa-Β ligand
TRAF6: Tumor necrosis factor receptor-related factor-6
F-actin: Filamentous actin
MCL: Micheliolide
DMAMCL: Dimethylamino micheliolide
NF-κB: Nuclear factor kappa-Β
PI3K: Phosphatidylinositol 3-kinase
MAPK: Mitogen-activated protein kinase
NFATc1: Nuclear factor of activated T cells-1
NLRP3: NOD-like receptor family pyrin domain-containing 3
GSDMD: Gasdermin D
IL-1β: Interferon 1β
TRAP: Tartrate-resistant acid phosphatase 5
BMM: Bone marrow-derived macrophage
ARS: Alizarin red S
BALP: Bone alkaline phosphatase
PINP: Procollagen type I N-propeptide
CTX-1: Collagen type I C-telopeptide
Ctsk: Cathepsin K
Dc-stamp: Dendritic cell-specific transmembrane protein
OIM: Osteogenic induction medium
Runx2: Runt-related transcription factor 2
COL 1: Type I collagen
OPN: Osteopontin
OCN: Osteocalcin
ALN: Alendronate
BV/TV: Bone volume/tissue volume
Tb.n: Trabecular number
Tb.Th: Trabecular thickness
Tb.pf: Trabecular pattern factor
