# Supplementary material for: ACT001 improves OVX-induced osteoporosis by suppressing the NF-κB/NLRP3 signaling pathway
Source: Mol Med. 2025 Apr 7;31:131. doi: 10.1186/s10020-025-01189-3 (PMC11977873; doi:10.1186/s10020-025-01189-3)
Supplement: Supplementary file 1 — Supplementary Material 1 [file 10020_2025_1189_MOESM1_ESM.docx]

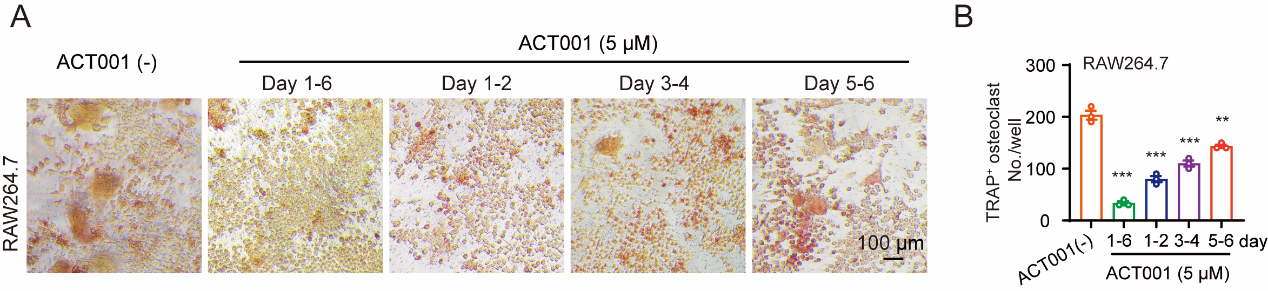


**Fig. S1** (A) RAW264.7 cells were induced by RANKL and treated with 5 μM ACT001 at different time point. The cells were subjected to TRAP staining and then visualized using a light microscope. Scale bar, 100 μm. (B) Quantitative statistics of the TRAP-positive multinucleated cells (nuclei ≥ 3). Data are presented as mean ± SEM. ** *p* < 0.01, *** *p* < 0.001.**x**


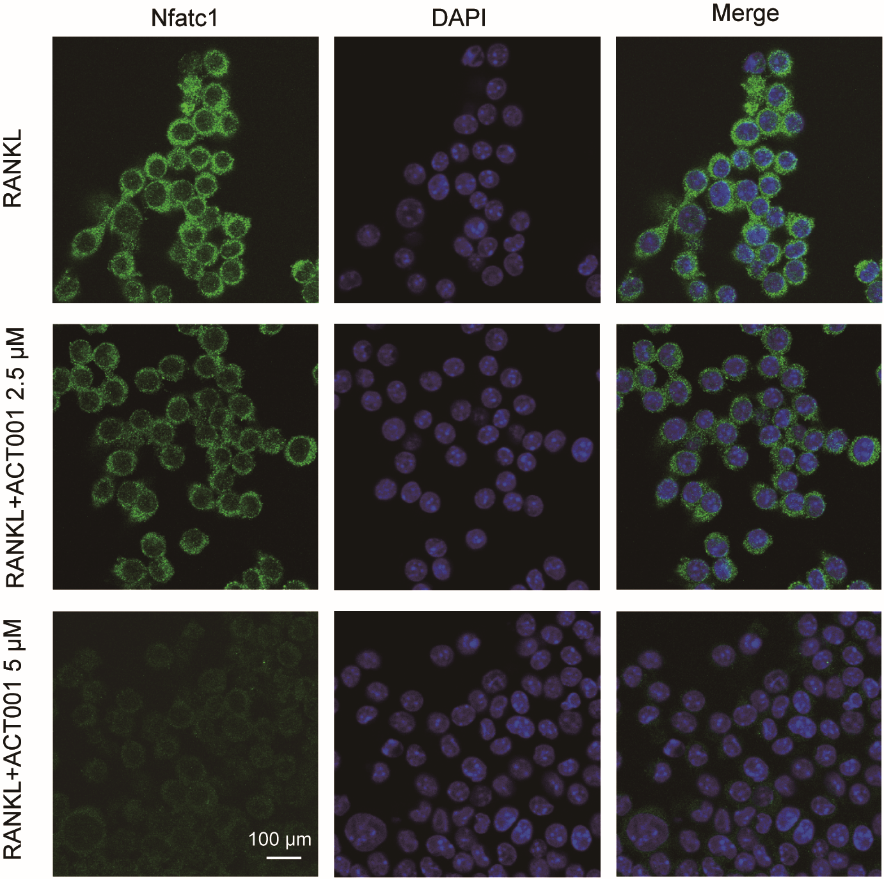


**Fig. S2** RAW264.7 cells were treated with RANKL in the presence of ACT001 (2.5, 5 μM) for 72 h, the expression of Nfatc1 were detected via immunofluorescence, Nfatc1 (green), and DAPI (blue). Scale bar, 100 μm.


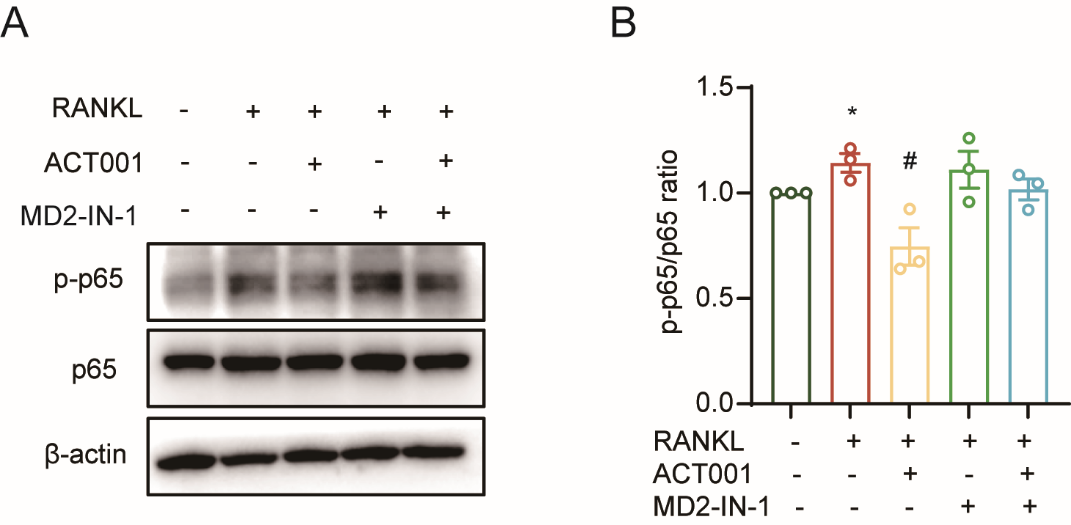


**Fig.S3** RAW264.7 cells were pretreated with MD2-IN-1 (10 μM) for 1 h, then incubated with or without ACT001 for another 2 h, followed by RANKL induction for 30 min, the protein expression levels of NF-κB pathway were detected via western blot. Data are presented as mean ± SEM. * *p* < 0.05 versus vehicle group, ^#^ *p* < 0.05 versus RANKL-treated group.


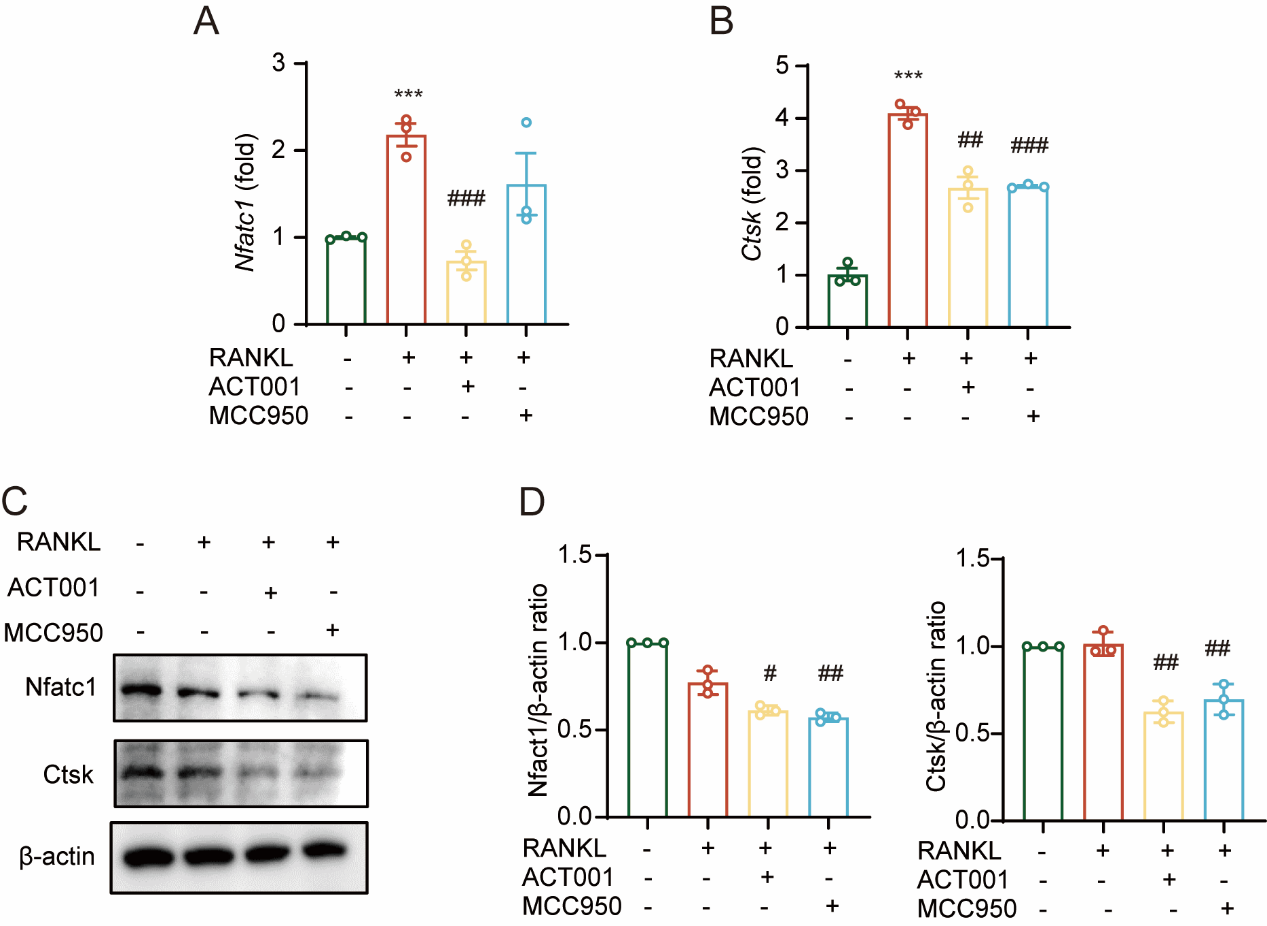


**Fig. S4** MCC950 inhibited RANKL-inducedd the activation of Nfatc1 and Ctsk. RAW264.7 cells were induced by RANKL and treated with 5 μM ACT001 or 5 nM MCC950 for 48 h. (A-B) The mRNA expression levels of Nfatc1 (A) and Ctsk (B) were detected via real-time PCR. (C) The protein expression levels of Nfatc1 and Ctsk were detected via western blot. (D) The relative ratio of the gray band value of the Nfatc1 and Ctsk to that of β-actin. Data are presented as mean ± SEM. *** *p* < 0.001 versus vehicle group. ^#^ *p* < 0.05, ^##^ *p* < 0.01, ^###^ *p* < 0.001 versus RANKL-treated group.


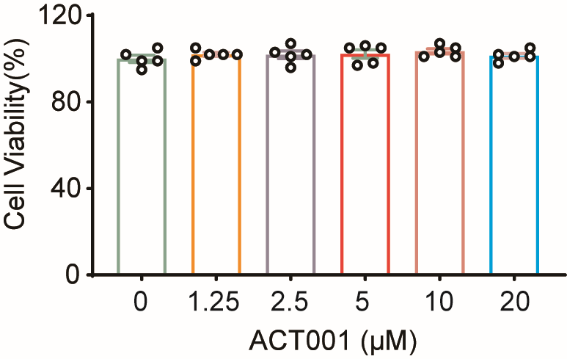


**Fig. S5** MC3T3-E1 cells were treated with different concentrations of ACT001 for 48 h, cell viability was detected using a CCK-8 assay.


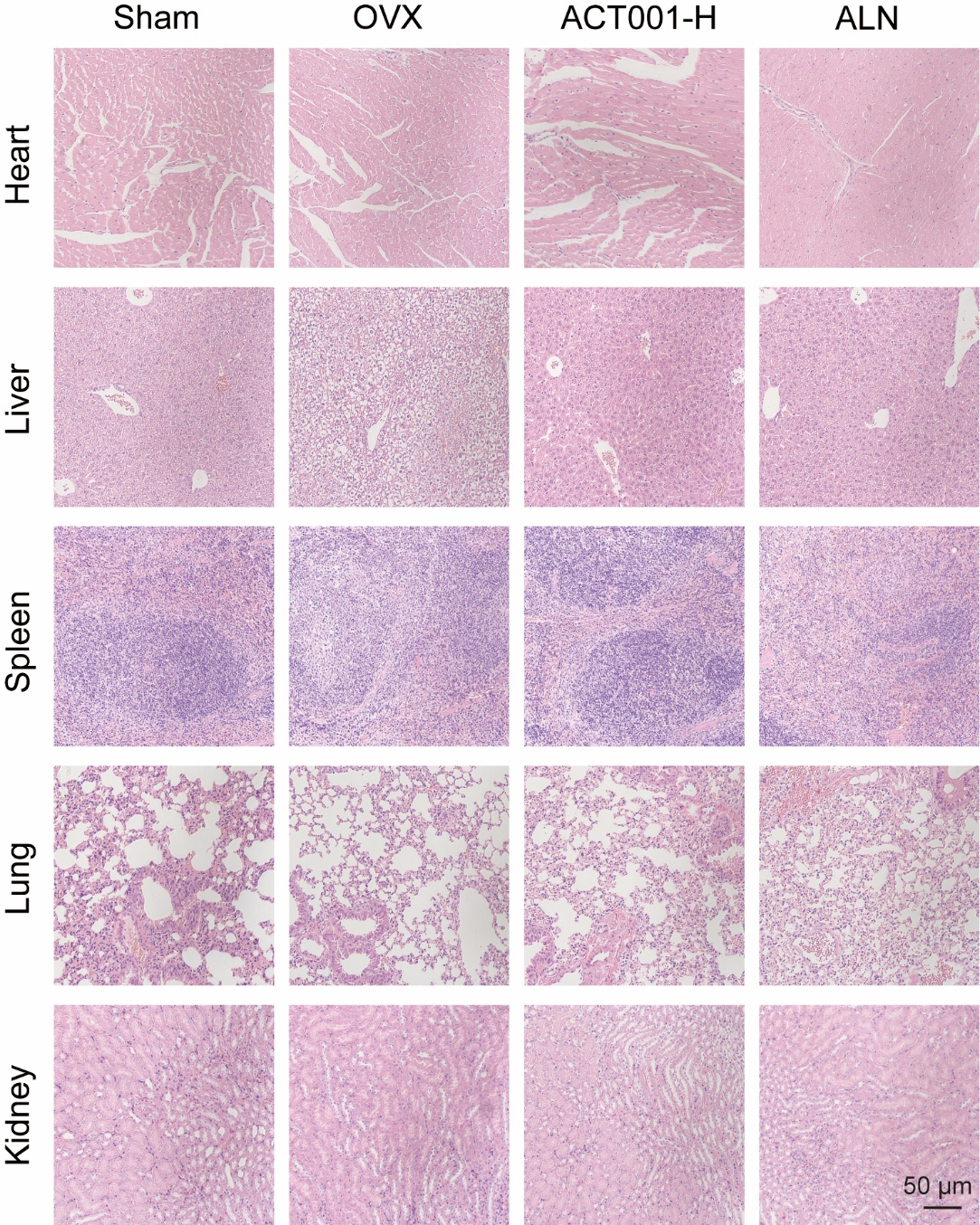


**Fig.S6** The H&E staining of major organs from the mice in each group (n=5).
